# Induction of aversive learning through thermogenetic activation of Kenyon cell ensembles in *Drosophila*

**DOI:** 10.3389/fnbeh.2014.00174

**Published:** 2014-05-15

**Authors:** David Vasmer, Atefeh Pooryasin, Thomas Riemensperger, André Fiala

**Affiliations:** Department of Molecular Neurobiology of Behavior, Johann-Friedrich-Blumenbach-Institute for Zoology and Anthropology, Georg-August-Universität GöttingenGöttingen, Germany

**Keywords:** *Drosophila melanogaster*, learning and memory, mushroom body, sparse coding, thermogenetics

## Abstract

*Drosophila* represents a model organism to analyze neuronal mechanisms underlying learning and memory. Kenyon cells of the *Drosophila* mushroom body are required for associative odor learning and memory retrieval. But is the mushroom body sufficient to acquire and retrieve an associative memory? To answer this question we have conceived an experimental approach to bypass olfactory sensory input and to thermogenetically activate sparse and random ensembles of Kenyon cells directly. We found that if the artifical activation of Kenyon cell ensembles coincides with a salient, aversive stimulus learning was induced. The animals adjusted their behavior in a subsequent test situation and actively avoided reactivation of these Kenyon cells. Our results show that Kenyon cell activity in coincidence with a salient aversive stimulus can suffice to form an associative memory. Memory retrieval is characterized by a closed feedback loop between a behavioral action and the reactivation of sparse ensembles of Kenyon cells.

## Introduction

**Figure 1:**
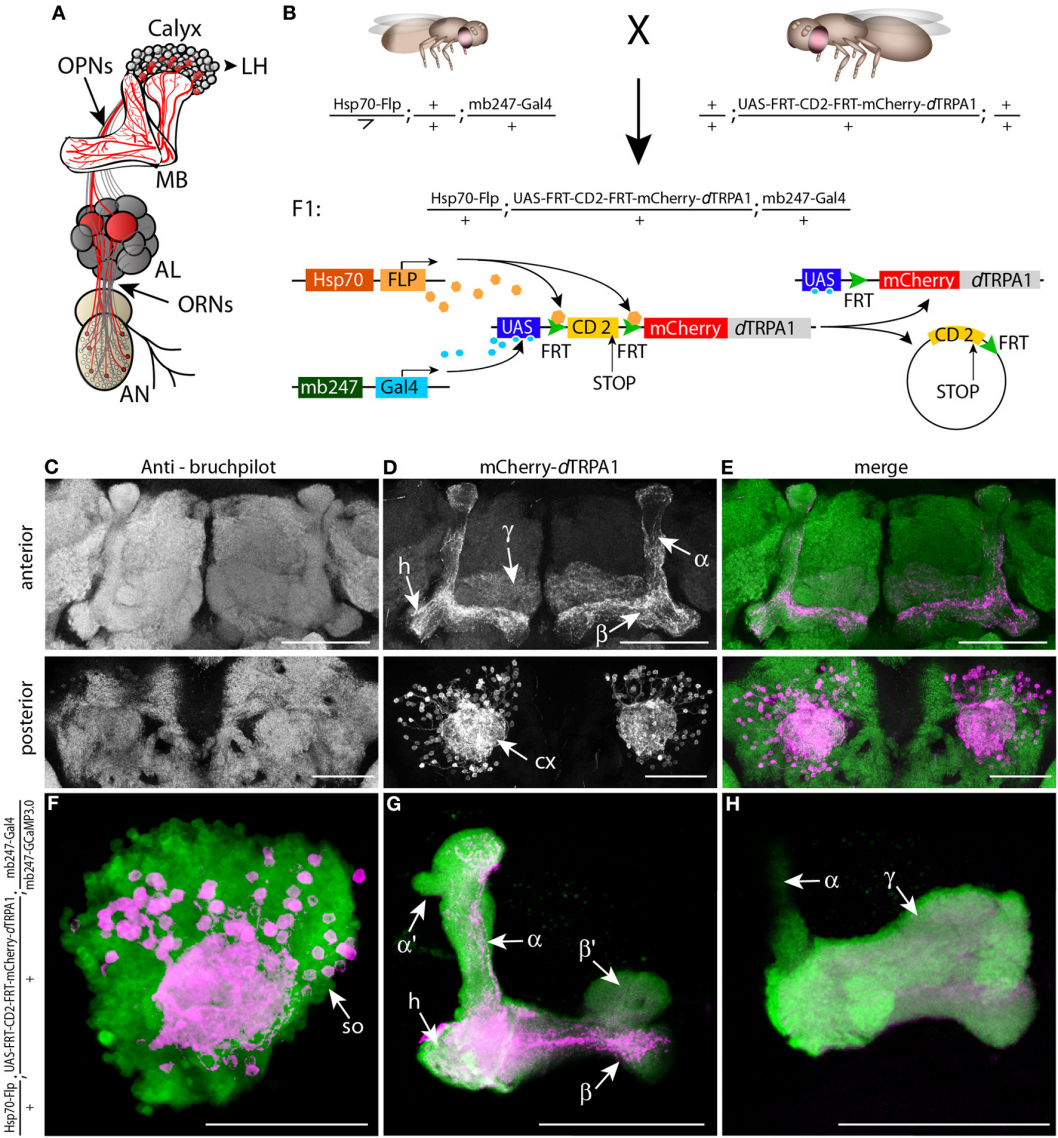
**mCherry-*d*TRPA1 expression in random ensembles of Kenyon cells. (A)** Illustration of the olfactory input to mushroom bodies in the *Drosophila* brain. Olfactory receptor neurons (ORNs) transmit odor information from the antennae (AN) to the antennal lobes (AL). Olfactory projection neurons (OPNs) connect the AL with the mushroom bodies (MB) at the calyx and the lateral horn (LH). **(B)** Principle of mCherry-*d*TRPA1 expression: a fly strain that carries the mushroom body-specific mb247-Gal4 DNA construct and a flippase DNA construct under control of a Hsp70 promoter is crossed to a fly strain that carries the mCherry-*d*TRPA1 DNA construct preceded by a FRT-flanked CD2(stop) cassette under UAS control. A flippase-mediated random excision of the FRT-cassette causes transcription of the mCherry-*d*TRPA1 construct. **(C–E)** Expression of mCherry-*d*TRPA1 in random ensembles of Kenyon cells. Upper panel anterior view, lower panel posterior view on a brain of a fly subjected to a 1 h heat shock 1 day after eclosion. Neuropils are visualized using the anti bruchpilot-antibody **(C)**, mCherry-*d*TRPA1 expression is visualized using an anti-RFP antibody **(D)**, and the overlay of both is shown in **(E)**. Scale bars = 50 μm. **(F–H)** Magnified illustration of the expression of mCherry-*d*TRPA1 in random ensembles of Kenyon cells visualized in magenta at the level of the somata (so). A large proportion of Kenyon cells express G-CamP3.0 as a fluorescence marker (green). **(F)** Kenyon cell axons that express mCherry-*d*TRPA1 and that project to the heel (h), the α- and β-lobes **(G)** and the γ-lobes **(H)**. Scale bars = 50 μm.

*Drosophila melanogaster* represents a key model organism for analyzing how neuronal circuits mediate associative learning and memory (Heisenberg, 2003; Davis, 2005; Fiala, 2007). Fruit flies can be trained to avoid an odor that has been presented in temporal coincidence with a punitive electric shock (Quinn et al., 1974; Tully and Quinn, 1985). The fruit fly perceives odors with ~1200 olfactory sensory neurons per hemisphere, located on the third antennal segments and maxillary palps (Vosshall and Stocker, 2007). Their axons converge in glomeruli of the antennal lobes, and ~150 olfactory projection neurons per hemisphere convey the odor information from the antennal lobes to the lateral horn and the calyces of the mushroom bodies (Vosshall and Stocker, 2007). Here, the olfactory projection neurons synapse onto Kenyon cells, the intrinsic neurons of the mushroom body (Figure 1A), where odors are encoded as sparsely activated ensembles of Kenyon cells (Perez-Orive et al., 2002; Murthy et al., 2008; Turner et al., 2008; Luo et al., 2010; Honegger et al., 2011). Odor stimuli activate ensembles of about 5% out of the ~2500 Kenyon cells per hemisphere, independently from the concentration or chemical complexity of the odorant (Honegger et al., 2011), and those ensembles are non-stereotypical and variable across individuals (Murthy et al., 2008).

A long line of evidence has demonstrated that Kenyon cells of the mushroom body are required for associative olfactory learning and the retrieval of short-term memory (Heisenberg et al., 1985; De Belle and Heisenberg, 1994; Connolly et al., 1996; Zars et al., 2000; Dubnau et al., 2001; McGuire et al., 2001; Heisenberg, 2003; Gerber et al., 2004; Davis, 2005; Fiala, 2007; Krashes et al., 2007; Qin et al., 2012). Structural mutations (Heisenberg et al., 1985) and ablations of mushroom bodies (De Belle and Heisenberg, 1994) or disruption of G-protein signaling in Kenyon cells (Connolly et al., 1996) impair olfactory learning, and blocking synaptic transmitter release from Kenyon cells impairs memory retrieval (Dubnau et al., 2001; McGuire et al., 2001). These experiments demonstrate the requirement of intact mushroom body function for associative olfactory learning, short-term memory formation, consolidation and memory recall. Moreover, gene mutations that affect learning, e.g., a mutation of the type I adenylate cyclase rutabaga (Zars et al., 2000), or of the D1-like dopamine receptor DopR (Qin et al., 2012), can be genetically rescued by expression of the wild type gene in subsets of Kenyon cells. The requirement of these gene products for olfactory learning can, therefore, be confined to the mushroom body. Altogether, these data have led to the hypothesis that the neuronal changes which mediate the behavioral changes caused by learning, i.e., the memory engram, can be allocated to the mushroom body (Heisenberg, 2003; Gerber et al., 2004; Fiala, 2007). All of these experiments are based on disruptive genetic, anatomical or physiological alterations of mushroom body function, or on the tissue-specific rescue of these alterations. It has never been tested, however, whether it is the activity of Kenyon cells in coincidence with a salient stimulus that is causative and sufficient to induce associative learning, an important criterion for localizing an essential memory trace or engram (Thompson, 2005; Riemensperger and Fiala, 2013). Alternatively, activity of projection neurons targeting the mushroom body and the lateral horn might be required for acquiring and retrieving an associative odor memory, and Kenyon cells might merely modulate the innate behavioral response elicited by these neurons. In this case, Kenyon cell activity would be required but not sufficient for olfactory learning. In order to provide this missing link we have conceived an experimental strategy to bypass any olfactory input to the mushroom body and to induce neuronal activity artificially in random ensembles of Kenyon cells in the absence of olfactory stimulation.

## Materials and methods

### *Drosophila* strains

Fly stocks were raised on standard cornmeal-agar food at 18°C, 60% humidity and a 12 h light-dark cycle. The wild type strain is Canton-S. The Hsp70-Flp strain (provided by G. Struhl) carrying an insertion on the X-chromosome is described in Basler and Struhl (1994). The mb247-Gal4 strain with an insertion on the third chromosome is described in Zars et al. (2000), the R71D08-Gal4 strain (provided by H. Tanimoto) with an insertion on the third chromosome has been described by Séjourné et al. (2011). G-Camp 3.0 (Tian et al., 2009) was expressed under control of two copies of the mb247 promoter (Zars et al., 2000) with an insertion on the third chromosome (Pech et al., 2013). For generating *d*TRPA1 (kindly provided by P. Garrity) tagged at the C-terminus mCherry was amplified using linker-PCR and inserted into the pUAST vector (Brand and Perrimon, 1993) using the restriction enzymes NotI and XhoI. The *d*TRPA1 cDNA from the pOX-*d*TRPA1 vector was amplified using linker-PCR and inserted into the pUAST-mCherry vector using the restriction enzymes BglII and MluI. For generating *d*TRPA1 tagged at the N-terminus mCherry was amplified using linker-PCR and inserted into the pUAST vector using the restriction enzymes BglII and NotI. The *d*TRPA1 cDNA was amplified using linker-PCR and inserted into the pUAS-mCherry vector using the restriction enzymes SpeI and XhoI. Flies carrying either version of the tagged *d*TRPA1 channel were tested for detectable fluorescence and for temperature-dependence of the channel by crossing the UAS lines to D42-Gal4 (Parkes et al., 1998) causing expression of the channel in motor neurons (Figure 6A). For expressing tagged *d*TRPA1 under control of mb247-Gal4 a line with mCherry at the C-terminus of *d*TRPA1 and an insertion on the X-chromosome was chosen because it showed no leaky expression though a high Gal4-dependent fluorescence when compared to other transgenic lines. Since several fly lines of both N- and C-terminal tagged *d*TRPA1 turned out to be functional, fluorescent and temperature-dependent (although with different efficiencies across lines with different P-element insertions) only the tag at the N-terminus of *d*TRPA1 was used to create the fly strain UAS-FRT-CD2(Stop)-FRT-mCherry-*d*TRPA1. The pUAST vector containing the FRT-CD2-y+-FRT cassette (Schlake and Bode, 1994) was provided by Gary Struhl. Part of the y+ sequence was cut out of the vector and an adaptor sequence with Acc65I, BsiWI, RSrII, and SacII restriction cutting sites was inserted downstream of the FRT cassette using the Acc65I and SacII restriction enzymes. The mCherry-*d*TRPA1 DNA sequence was amplified using linker-PCR and inserted into the UAS-FRT-CD2(Stop)-FRT vector using the restriction enzymes BsiWI and SacII. Germline transformation was performed commercially (BestGene Inc.). A fly line with an insertion on the second chromosome was chosen for further studies because of a relatively high fluorescence level. The Hsp70-FLP insertion on the X-chromosome was combined with the mb247-Gal4 insertion on the third chromosome to generate a Hsp70-FLP;+;mb247-Gal4 strain homozygous for both P-element inserts. Males of this fly strain were crossed to virgin females of UAS-FRT-CD2(Stop)-FRT-mCherry-*d*TRPA1 flies with the insertion balanced over CyO. Female offspring of this cross younger than 2 days were anaesthetized using CO_2_ and transferred into fresh food vials. To induce FLP-mediated expression flies were incubated at 30°C for 1 h unless otherwise indicated.

### Immunohistochemistry

Brains and, in some cases, thoracic ganglia, were dissected in ice-cold Ringer's solution and fixed for 2 h on ice in 4% paraformaldehyde dissolved in phosphate buffered saline (PBS), followed by three washing steps in PBS containing 0.5% Triton X-100 (PBST) for 20 min each. After 2 h of preincubation in PBST containing 2% bovine serum albumin (block solution) brains were incubated for 24 h at 4°C with the primary antibodies diluted in block solution. The following antibodies were used: mouse anti-nc82 against Bruchpilot (Wagh et al., 2006) (provided by Erich Buchner) diluted 1:10, rat anti-RFP (5F8, Chromotec) to stain mCherry diluted 1:300, and rabbit anti-GFP (A6455, Invitrogen) diluted 1:200. Subsequently, brains were washed three times for 20 min each in PBST and incubated over night with the secondary antibodies at 4°C. The following secondary antibodies were used: goat anti-mouse conjugated with Alexa Fluor 488 (A1101, Invitrogen), goat anti-rabbit conjugated with Alexa Fluor 488 (A11034, Invitrogen) and goat anti-rat conjugated with Cy3 (A10522, Invitrogen), all diluted 1:500. Brains were washed three times in PBST for 20 min each, washed in PBS overnight at 4°C and embedded in Vectashield (Vector Laboratories). Images were acquired using a confocal laser scan microscope (Leica) and analyzed using ImageJ. For 3D-reconstructions Amira 5.3.3 software (Visage Imaging) was used. The strength of mCherry expression was quantified in an area determined by a focal plane that covered a part of the mushroom body peduncle in which α/β- and γ-lobes were identifiable (Sinakevitch et al., 2010). The areas of the respective regions were determined using anti-bruchpilot immunereactivity and the relative proportion of anti-RFP immuneractivity indicating mCherry-*d*TRPA1 was quantified. Mushroom bodies of both hemispheres were examined when possible. For quantitative image analysis the Amira 5.3.3 (Visage Imaging) and ImageJ software was used.

### Behavioral analysis

For training eight female flies (4–6 days old) were transferred into tubes that were covered inside with an electrifiable copper grid. The tubes were pre-warmed to 30°C and training was performed in an illuminated incubator at 30°C and an air humidity of ~60%. Animals were kept in these tubes for 2 min and 24 electric shocks of 90 V DC and 1.25 s duration were applied with 3.75 s breaks, resulting in 5 s intervals. For the “unpaired” training paradigm flies were treated accordingly but with a 5 min delay between shock application and artificial activation of Kenyon cell subsets (Figure 7A). In the “forward” training paradigm flies were incubated at 30°C and subsequently electric shocks were applied, whereas in “backward” training the flies were first exposed to 2 min of electric shocks on 18°C and were subsequently transferred for 2 min to 30°C (Figures 7B,C). Control animals were either treated equally but without receiving electric shocks or were trained at 18°C. Subsequently, flies were transferred to a heat-gradient chamber that consisted of an aluminum block with 8 walking tracks (225 mm length × 5 mm width × 4 mm height) covered with a Plexiglas lid. The aluminum block was connected at both ends with water-cooled and electronically controlled Peltier elements. The entire apparatus was kept in an incubator at constant white light, 16°C temperature and ~65% humidity. The Peltier elements produced a linear and stable temperature gradient over the length of the arenas ranging from 18 to 35°C. Individual flies were carefully transferred without anesthesia into the walking tracks through small holes in the lid and could distribute freely for 20 min. The walking traces were monitored from above with a high definition video camera (Panasonic HC-V500). For data analysis, flies were tracked using the Noldus EthovisionXT 8.5 software (Wageningen). Temperature preferences were determined as the average position of each fly within the last 5 min of the observation period and the position of each fly within the chamber was assigned to an exact temperature value. Immediately after the behavioral experiments random samples of flies were anesthetized, brains were dissected and mCherry-*d*TRPA1 expression was determined using immunehistochemistry. To test for reactivity and avoidance of electric shocks groups of 20–30 flies were tested in a T-maze apparatus consisting of two tubes covered inside with an electrifiable copper grid, one of which was electrified for 1.25 s at 90 V in 5 s intervals for 2 min. Animals could distribute freely between the two tubes and the numbers of animals in the electrified tube (N_*shock*_) and non-electrified tube (N_*no shock*_) were counted. An avoidance index (AI) was calculated as AI = (N_*shock*_ − N_*no shock*_)/(N_*shock*_ + N_*no shock*_). A negative AI value indicates avoidance of the electrified tube.

## Results

### Expression of mcherry-tagged *d*TRPA1 in random ensembles of mushroom body kenyon cells

**Figure 2:**
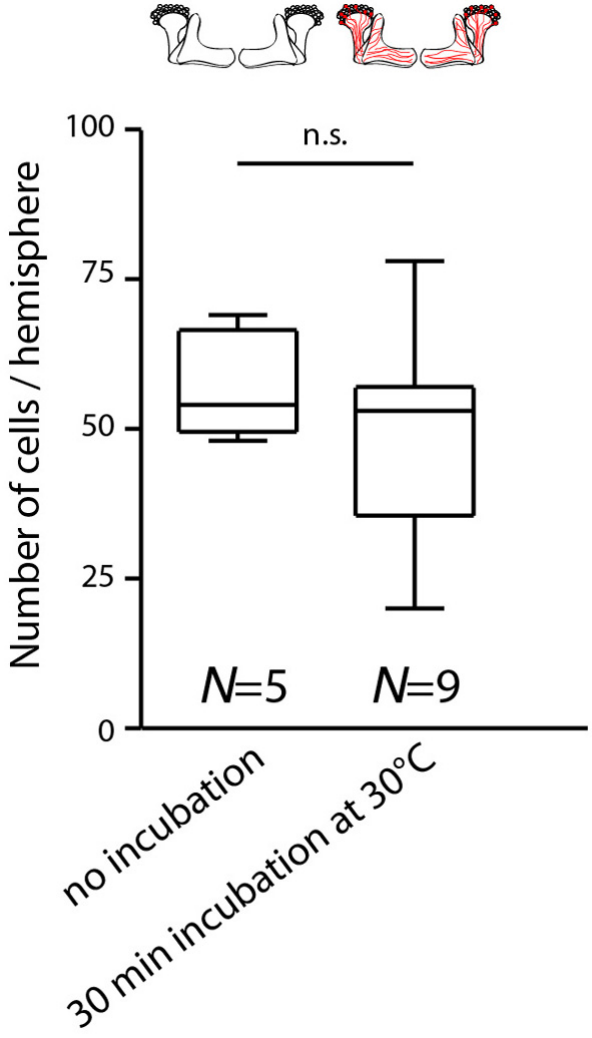
**Temperature increase during behavioral experiments does not rapidly affect the expression of mCherry-*d*TRPA1 in Kenyon cells**. Flies expressing mCherry-*d*TRPA1 in random subset of Kenyon cells were either raised and maintained at 18°C or raised at 18°C and transiently incubated for 30 min at 30°C at an age of 4–6 days. The duration of the elevated temperature matches the maximum time that animals can spend at temperatures above 25°C during all behavioral experiments. Subsequently the brains were dissected and the numbers of mCherry-*d*TRPA1 expressing Kenyon cells determined. Flies incubated at 18°C showed mCherry-*d*TRPA1 expression in 56.9 ± 8.1 (mean ± SD) Kenyon cells, whereas flies exposed for 30 min to 30°C showed expression in 48.0 ± 16.8 (mean ± SD) Kenyon cells (*p* > 0.2, Mann-Whitney-U-Test).

The thermo-inducible cation-channel *d*TRPA1 (Hamada et al., 2008) was fused with the red fluorescent protein mCherry (Shaner et al., 2005) and placed downstream of a stop cassette flanked by two flippase recognition target (FRT) sequences (Schlake and Bode, 1994) (Figure 1B). Transgenic flies that carry this construct together with a flippase under control of a heat shock promoter (Basler and Struhl, 1994) were generated for expressing mCherry-*d*TRPA1 in random subpopulations of neurons (Figure 1B). Using this technique in combination with the mb247-Gal4 driver line (Zars et al., 2000) we obtained mosaic flies that expressed mCherry-*d*TRPA1 in random subsets of α/β- and γ-lobe Kenyon cells (Figures 1C–H). Those neurons that express *d*TRPA1 can be depolarized by raising the temperature above ~25°C in adult flies (Hamada et al., 2008; Tang et al., 2013). Since both the induction of gene expression by a heat shock promoter during development and the artificial, thermogenetic activation of Kenyon cells during the behavioral experiment are temperature-dependent processes, we first tested and confirmed that the maximal duration of the behavioral experiments (<30 min) was not sufficient to induce any rapid additional gene expression during the behavioral tests (Figure 2).

### Thermogenetic induction of associative learning

**Figure 3:**
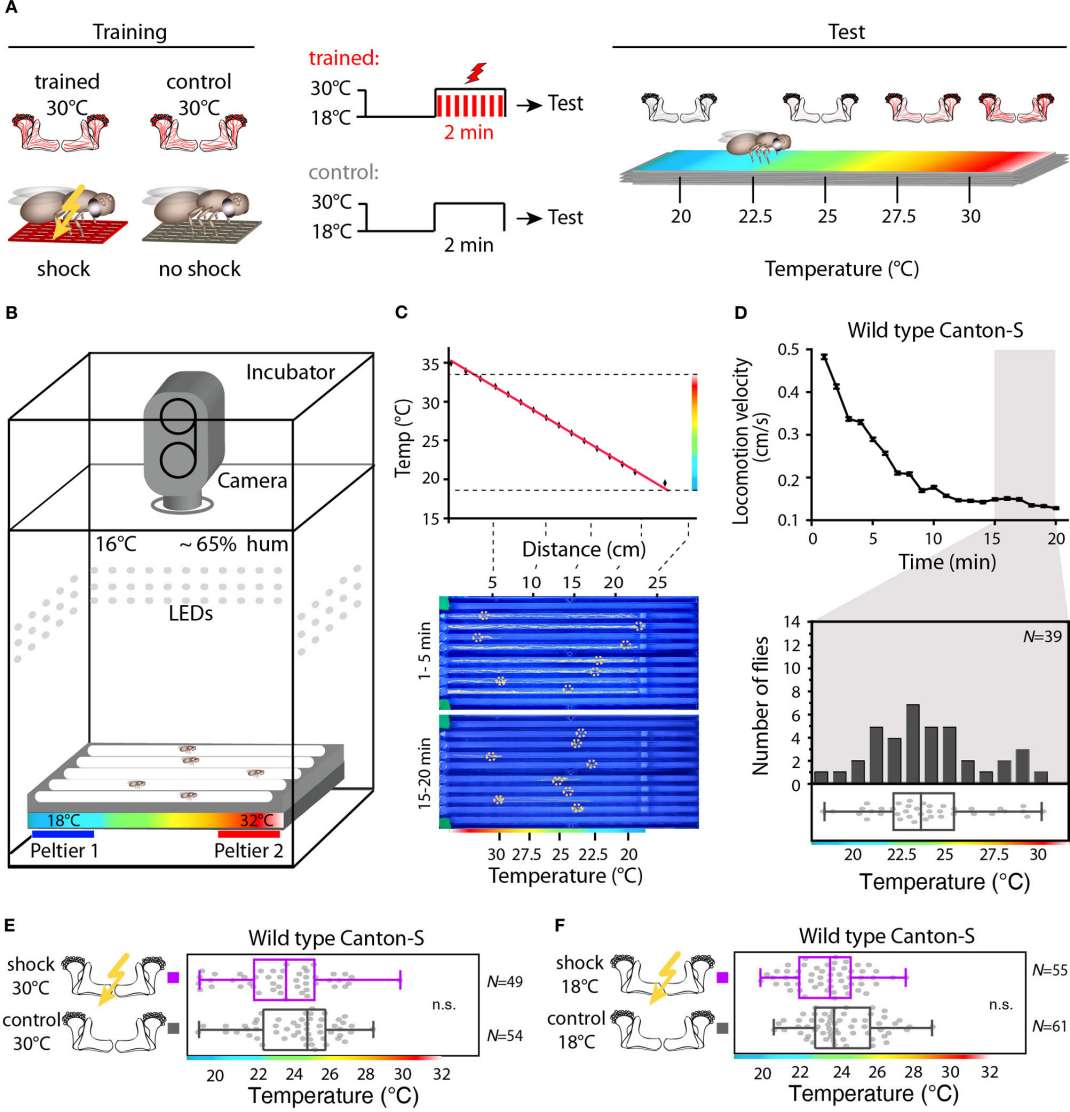
**Illustration of the training and temperature preference test. (A)** Flies are exposed to 30°C to thermogenetically activate Kenyon cells for 2 min simultaneously with electric shocks (“Training”). A second group of animals is treated equally, but without electric shocks. Subsequently, flies are individually transferred into a temperature gradient arena (“Test”). **(B)** A metal block with eight walking tracks covered with a Plexiglas lid is equipped at the ends with Peltier elements. The flies can freely distribute for 20 min within each track at 65% air humidity and white light conditions and their locomotion is monitored using a video camera. **(C)** Upper panel: the Peltier elements create a linear temperature gradient from 18 to 35°C. The red line indicates a linear fit (*R*^2^ = 0.99). Lower panel: snapshots of the temperature readout arena indicating positions and locomotion traces of individual flies during the first 5 min (upper part) and during the last 5 min (lower part) of the 20 min observation time. **(D)** Upper panel: locomotion velocity of naïve wild type flies. Temperature preferences are determined during the last 5 min of the observation period (gray bar), when exploratory behavior is minimal. Lower panel: the bars in the upper part of the graph show the numbers of naïve wild type flies with a preference for the temperature range in 1°C bins indicated on the x-axis. The dots in the lower part indicate temperature preferences for all individual flies. The superimposed lines indicate median and IQR. Whiskers indicate minimum and maximum values. **(E)** Temperature is not learned as a conditioned stimulus. Temperature preference of wild type Canton-S flies that were incubated for 2 min at 30°C and simultaneously received electric shocks (“trained”) is not significantly different (*p* > 0.1) from that of flies incubated at 30°C, but without electric shocks (“control”). **(F)** Wild type Canton-S flies that were trained accordingly, but were incubated for 2 min at 18°C and simultaneously received electric shocks (“trained”) and flies that have been equally treated, but without electric shocks (“control”) did not show any significant difference (*p* > 0.05) in their temperature preference.

**Figure 4:**
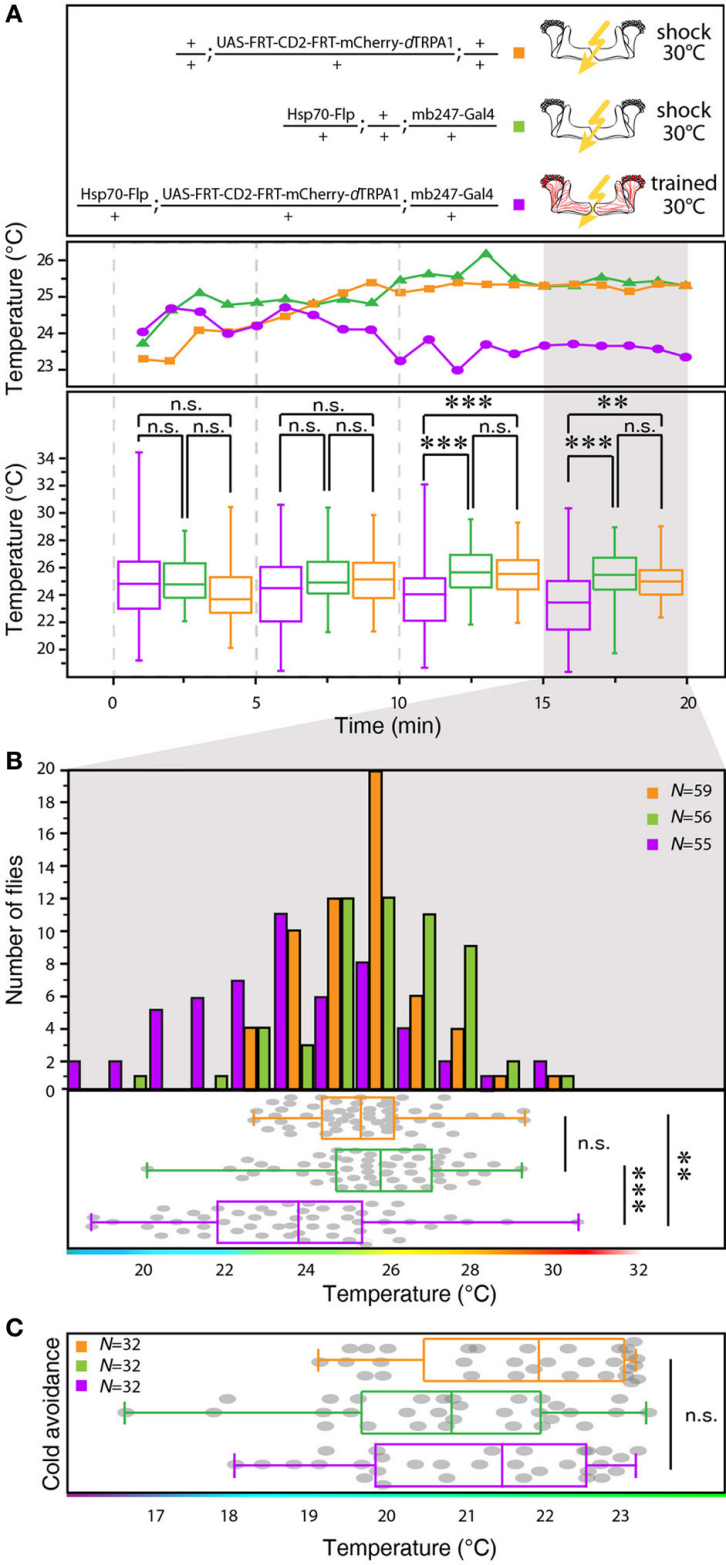
**Between-genotype comparison of mCherry-*d*TRPA1 expressing flies and genetic control animals. (A)** Flies expressing mCherry-*d*TRPA1 in random ensembles of Kenyon cells show a shift in temperature preference when trained at 30°C for 2 min simultaneously with electric shocks compared to heterozygous control flies carrying the UAS-FRT-CD2(stop)-FRT-mCherry-*d*TRPA1 construct and heterozygous control flies carrying the Hsp70-Flp and the mb247-Gal4 constructs. The traces show the median temperature preference over time in 60 s bins during the 20 min test phase. The lower part of the graph shows temperature preferences in 5 min bins during the 20 min test phase. A significant change in temperature preferences is observed after ~10 min. Box plots indicate medians and interquartile ranges, minimum and maximum values. **(B)** Temperature preference of each genotype within the last 5 min of the 20 min test phase. Bars indicate the numbers of flies with a preference for the temperature range in 1°C bins indicated on the x-axis. The dots indicate temperature preferences of all individual flies. The superimposed lines indicate medians and interquartile ranges. Whiskers indicate minimum and maximum values. **(C)** After training at 30°C flies expressing mCherry-*d*TRPA1 in random ensembles of Kenyon cells no significant difference in avoidance of cool temperatures is observed between genotypes. Statistical test: Kruskal-Wallis-test with Bonferroni-corrected *post-hoc* Mann-Whitney-U-test (^**^p < 0.01; ^***^p < 0.001).

**Figure 5:**
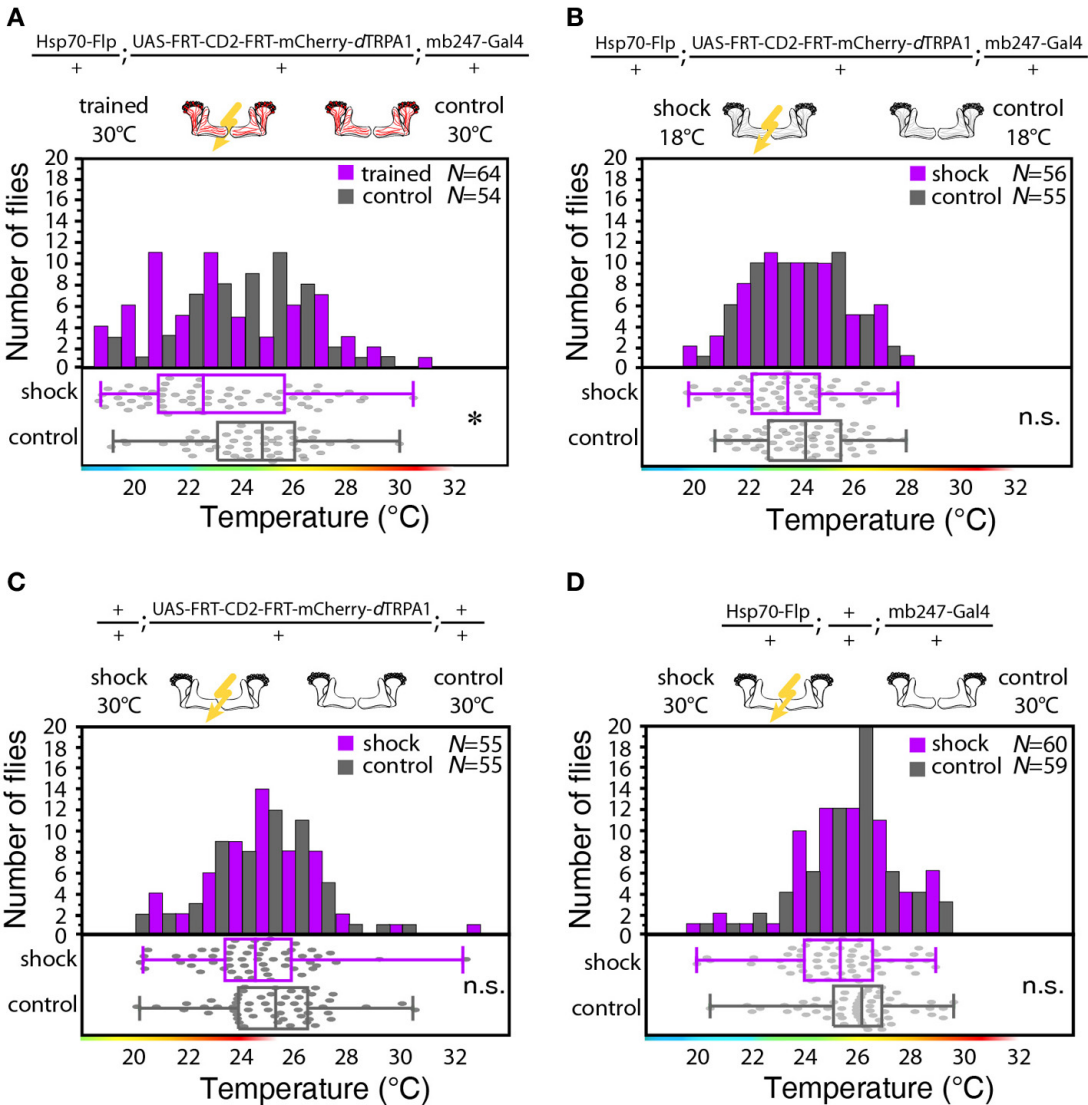
**Thermogenetic induction of learning. (A)** Temperature preferences of trained and control flies that express mCherry-*d*TRPA1 in random ensembles of Kenyon cells. Flies trained at 30°C show a significant (*p* < 0.02) shift in temperature preference compared to control animals, i.e., animals that did not receive electric shocks. **(B)** Temperature preference of flies that express mCherry-*d*TRPA1 in random ensembles of Kenyon cells treated with or without electric shocks at 18°C. No significant (*p* > 0.08, Mann-Whitney-U-test) difference in the temperature preference between trained flies and control flies is detectable. **(C)** Temperature preference of flies heterozygous for the UAS-FRT-CD2(stop)-FRT mCherry-*d*TRPA1 DNA construct treated with or without electric shocks at 30°C. No significant (*p* > 0.2) differences in the temperature preference between trained flies and control flies is detectable. **(D)** Temperature preference of flies heterozygous for the Hsp70-Flp and the mb247-Gal4 DNA constructs treated with or without electric shocks at 30°C. No significant (*p* > 0.05) differences in the temperature preference between trained flies and control flies is detectable. In panels **(A–D)** bars indicate the numbers of flies with a preference for the temperature range indicated on the x-axis in 1°C bins. The dots indicate temperature preferences of all individual flies. The superimposed lines indicate medians and interquartile ranges. Whiskers indicate minimum and maximum values. Statistical test: Mann-Whitney-U-test with Bonferroni correction. (^*^p < 0.05).

**Figure 6:**
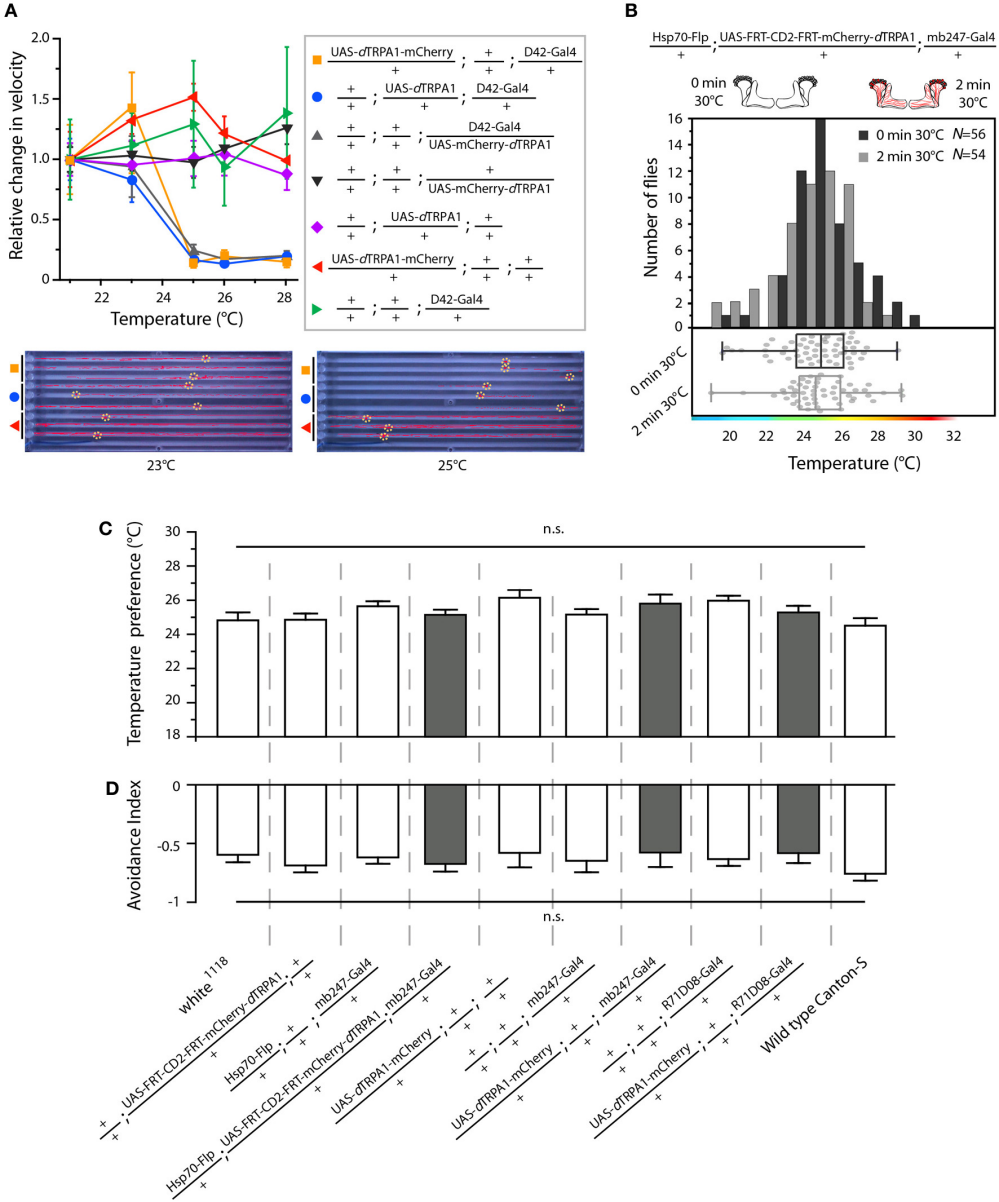
**Functional characterization of mCherry-tagged *d*TRPA1 expression. (A)** *d*TRPA1 tagged with mCherry at either N- or C-terminus were expressed in motor-neurons using D42-Gal4 (Parkes et al., 1998) and locomotion was observed at different temperatures in comparison to flies expressing untagged *d*TRPA1 (Hamada et al., 2008). Flies expressing either form of *d*TRPA1 in motor-neurons showed similar locomotion impairments at temperatures above ~25°C, whereas genetic control strains did not. Lower part: snapshots of the temperature readout arena indicating positions and locomotion traces of individual flies during 5 min at 23°C (left) and 25°C (right). **(B)** Thermogenetic activation of random ensembles of Kenyon cells in flies exposed to 30°C for 2 min did not influence subsequent temperature preference when compared to naïve animals that were not exposed to 30°C (*p* > 0.8, Mann-Whitney-U-test). Bars indicate the numbers of flies with preferences for the temperature range in 1°C bins indicated on the x-axis. The dots indicate temperature preferences of all individual flies. The superimposed lines indicate medians and interquartile ranges. **(C)** Naïve temperature preferences were not affected in fly strains expressing mCherry-tagged *d*TRPA1 in random Kenyon cell ensembles, in a large proportion of Kenyon cells under control of mb247-Gal4 (gray bars) or in a subset of mushroom body output neurons when compared to genetic control strains or wild type flies (white bars). (*p* > 0.06, Bonferroni-corrected One-Way ANOVA, *N* ≥ 24). **(D)** There was no significant difference between the same fly strains in the avoidance of electric shocks at 30°C (*p* > 0.07, Bonferroni-corrected One-Way ANOVA, *N* ≥ 5). Bars indicate means ± s.e.m.

We designed a learning paradigm in which the artificial, thermogenetic activation of Kenyon cell ensembles was temporally paired with electric shocks. Flies were trained in tubes covered inside with electric grids at 30°C, i.e., at a temperature well above the temperature required for thermogenetic activation of neurons using *d*TRPA1 (Hamada et al., 2008) for 2 min. Simultaneously with the thermogenetic activation of random Kenyon cell ensembles electric shocks of 90 V (1.25 s shock duration with 3.75 s intervals) were applied. Control animals of the same genotype were treated equally, but did not receive any electric shocks (Figure 3A). In the typical aversive olfactory conditioning procedure the animals learn to avoid the odor that has been temporally paired with the punishment (Tully and Quinn, 1985). We reasoned that if the Kenyon cells' activity can provide a neuronal manifestation of a memory engram the animals should avoid any reactivation of the trained Kenyon cell ensembles. To test this hypothesis, directly after the training procedure the animals were individually transferred into a test chamber in which they could walk freely along a temperature gradient ranging from 18 to 35°C (Figures 3B–D). The locomotion of each animal was monitored and the temperature preference was interpreted as memory readout, whether the animals approached or avoided the activation of those Kenyon cells expressing mCherry-*d*TRPA1, i.e., temperatures above ~25°C. To exclude the possibility that the animals would learn to associate a temperature sensation as a conditioned stimulus with the punishment, we first subjected wild type Canton-S flies, to either 30 or 18°C and applied electric shocks simultaneously. In the test situation, no significant differences in these flies' temperature preferences were detectable when compared to animals that did not receive an electric shock (Figures 3E,F). This demonstrates that flies do not associate the elevated temperature per se with the electric shock punishment. However, flies that expressed mCherry-*d*TRPA1 in random subsets of Kenyon cells and that had received electric shocks simultaneous to the thermogenetic induction of neuronal activity showed a significant shift toward lower temperatures in the test situation (median 23.6°C, 3.5°C interquartile range, IQR) when compared to genetic control flies, (median 25.6°C, 2.3°C IQR for Hsp70-FLP;+;mb247-Gal4 and 25.1°C, 1.8°C IQR for UAS-FRT-CD2(Stop)-FRT-mCherry-*d*TRPA1) (Figures 4A,B), but no significant alteration in cold avoidance (Figure 4C). We further compared flies of the same genotype that have either received electric shocks simultaneous to 30°C temperature exposure or that were exposed to 30°C alone. Again, flies expressing mCherry-*dTRPA1* in random subsets of Kenyon cells showed a significant shift toward lower temperatures when treated with CS and US simultaneously compared to control flies (Figure 5A). Heterozygous genetic controls, however, did not show a difference in their temperature preference behavior between trained and control flies (Figures 5C,D). As an additional control, flies expressing mCherry-*d*TRPA1 in subsets of Kenyon cells were subjected to training with electric shocks at 18°C, i.e., without any artificial activation of Kenyon cell ensembles. No significant difference between flies trained at 18°C and control flies that did not receive any electric shock treatment was observed (Figure 5B). The *d*TRPA1 channel starts opening at ~25°C (Hamada et al., 2008; Tang et al., 2013) (Figure 6A), i.e., in the range of the preferred temperature of naïve fruit flies (Sayeed and Benzer, 1996; Hamada et al., 2008) (Figures 3D, 6C). Therefore, the learned shift in temperature preference reflects that the trained animals actively prevent a reactivation of Kenyon cells. To test whether the observed change in temperature preference of flies expressing mCherry-*d*TRPA1 in Kenyon cell subsets is due to a sensitization of the animals we thermogenetically activated Kenyon cell ensembles for 2 min only and tested them subsequently in comparison to naïve animals. No changes in the temperature preference between the two groups of animals could be detected (Figure 6B), which demonstrates that activating subsets of Kenyon cells did not merely sensitize the animals to avoid warmer temperatures. Furthermore, no significant differences in temperature preference or electric shock reactivity in naïve flies expressing *d*TRPA1-mCherry in a major part of the mushroom bodies using mb247-Gal4 or in mosaic-flies expressing mCherry-*d*TRPA1 in random subsets of Kenyon cells were observed when compared to the genetic control strains and to wild type flies (Figures 6C,D).

### Learned association of activated kenyon cell ensembles with electric shocks is dependent on the temporal coincidence of both stimuli

**Figure 7:**
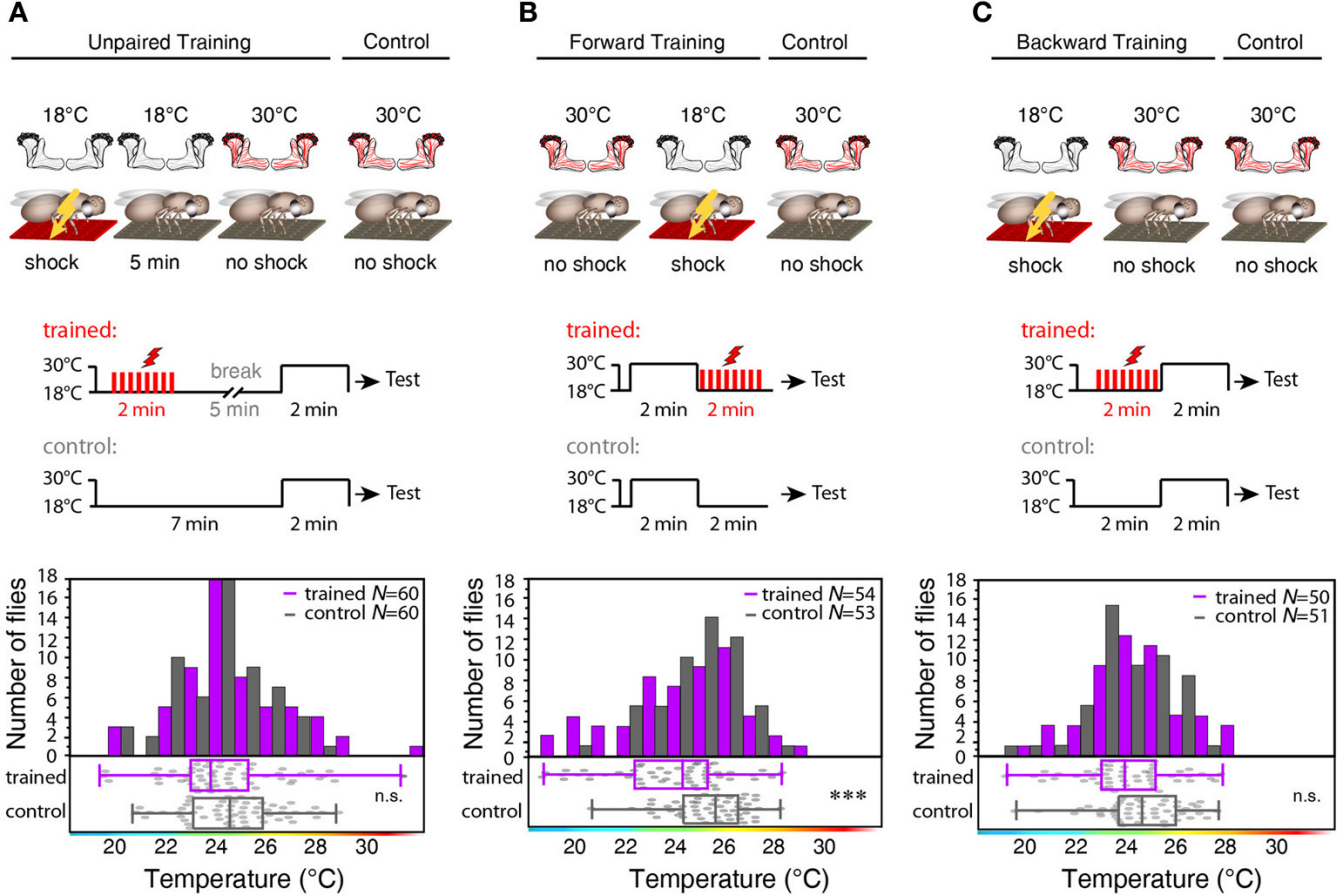
**Forward, but not backward or unpaired training causes learning. (A)** “Unpaired” training paradigm. Flies expressing mCherry-*d*TRPA1 in random ensembles of Kenyon cells were subjected for 2 min to electric shocks and, after a 5 min delay, exposed to 30°C to activate Kenyon cells (“Training”). Control animals were treated equally, but without electric shocks. No significant (*p* > 0.1) difference in temperature preference was observed between trained animals and control animals. Bars indicate the numbers of flies with preferences for the temperature range in 1°C bins indicated on the x-axis. The dots indicate temperature preferences of all individual flies. The superimposed lines indicate medians and interquartile ranges. **(B)** “Forward” training paradigm. Flies expressing mCherry-*d*TRPA1 in random ensembles of Kenyon cells were incubated at 30°C to activate Kenyon cells and subsequently subjected for 2 min to electric shocks. Control animals were treated equally, but without electric shocks. Trained flies showed a significant (*p* < 0.001) shift in temperature preference compared to control animals. **(C)** “Backward training” paradigm. Flies expressing mCherry-*d*TRPA1 in random ensembles of Kenyon cells were subjected for 2 min to electric shocks and subsequently exposed to 30°C to activate Kenyon cells. Control animals were treated equally, but did not receive any electric shocks. Trained flies did not show any significant (*p* > 0.05) shift in temperature preference compared to control animals. Statistical test: Mann-Whitney-U-test (^***^p < 0.001).

To test whether the training-induced change in behavior is indeed caused by a temporal coincidence or contiguity of Kenyon cell activity and punishment we temporally separated thermogenetic neuronal activation and electric shock by 5 min (”unpaired training”). No significant shift in the temperature preference of treated and control flies that did not receive any electric shocks could be observed (Figure 7A). To further test whether the temporal sequence of the artificially induced Kenyon cell ensembles and the punishment affects learning, we subjected the animals also to a “forward” and a “backward” training paradigm. Flies indeed showed a significant change in behavior, when the thermogenetic activation of Kenyon cells preceded the subsequent punishment (“forward training,” Figure 7B). On the contrary, “backward training” during which the punishment preceded the Kenyon cell activation did not cause any change in behavior (Figure 7C). These experiments demonstrate that it is indeed the temporal contiguity between Kenyon cell activity and punishment that causes the formation of an aversive memory. In conclusion, input to the mushroom body is not required for forming an associative memory, and the activity of the trained Kenyon cells drives the learned avoidance behavior.

### Thermogenetic neuronal activation downstream of kenyon cells does not induce learning

**Figure 8:**
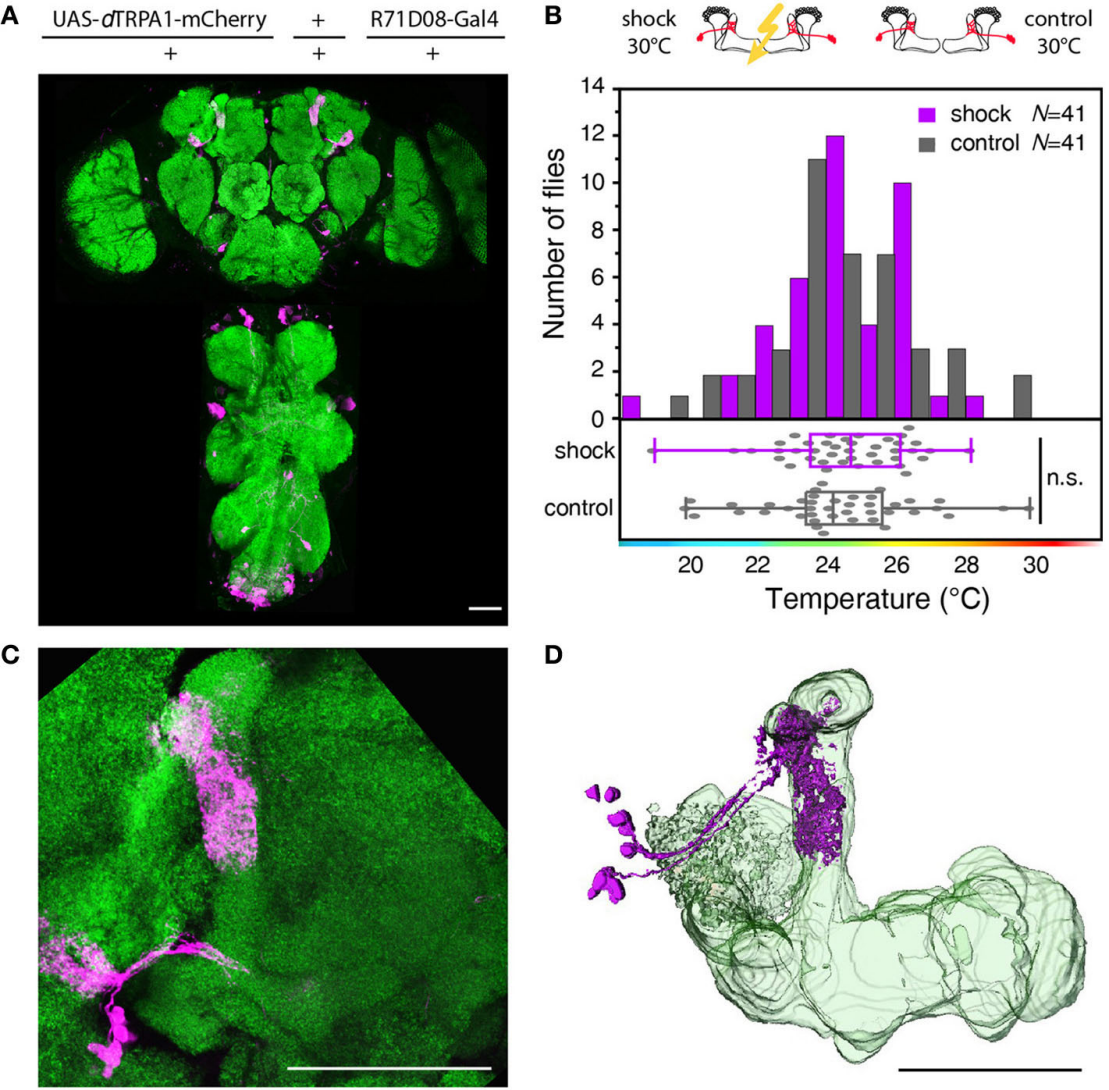
**Artificial activation of mushroom body output neurons temporally paired with electric shock does not cause learning. (A)** *d*TRPA1-mCherry expression driven by R71D08-Gal4 in the brain and thoracic ganglion. **(B)** Temperature preferences of trained and control flies that express *d*TRPA1-mCherry in mushroom body output neurons. Bars indicate the numbers of flies with a preference for the temperature range in 1°C bins indicated on the x-axis. The dots indicate temperature preferences for individual flies. The superimposed lines indicate medians and interquartile ranges. Flies trained at 30°C showed no significant shift in temperature preference compared to controls (*p* > 0.4; Mann-Whitney-U-test). **(C)** Magnified illustration of the *d*TRPA1-mCherry expression in a population of mushroom body output neurons determined by R71D08-Gal4. **(D)** 3D reconstruction of R71D08-Gal4 positive mushroom body output neurons. Neuropils stained with anti-bruchpilot antibody are depicted in green and *d*TRPA1-mCherry expression stained with anti-RFP antibody in magenta. Scale bars = 50 μm.

Next, we tested whether learning can be localized to neurons downstream from Kenyon cells (mushroom body output neurons). We focused on those mushroom body output neurons that have been shown to be required for memory retrieval (Séjourné et al., 2011). These mushroom body output neurons have been shown to respond to olfactory stimuli (Séjourné et al., 2011). Temporal pairing of an odor with an electric shock punishment causes a reduction of Ca^2+^ activity elicited by the trained odor, but not by a control odor after learning. As these neurons respond strongly to odor stimuli we tested whether the activity of these neurons in coincidence with an electric shock during training can elicit learning downstream from the mushroom bodies. We expressed *d*TRPA1-mCherry in these neurons and some unidentified neurons in the thoracic ganglia using the R71D08-Gal4 fly strain (Figures 8A,C,D), and presented electric shocks simultaneously with thermogenetic neuronal activation. We could not observe significant behavioral changes in the test situation (Figure 8B). While these output neurons have been shown to be necessary to decode a memory (Séjourné et al., 2011), we have found that thermogenetic activation of those neurons in coincidence with the punishment is not sufficient for memory acquisition or retrieval. Of course, it cannot be excluded that an artificial activation of other types or more neurons downstream from Kenyon cells in coincidence with a punitive stimulus might cause a learned association.

### Sparseness of activated kenyon cell ensembles is critical for associative learning

**Figure 9:**
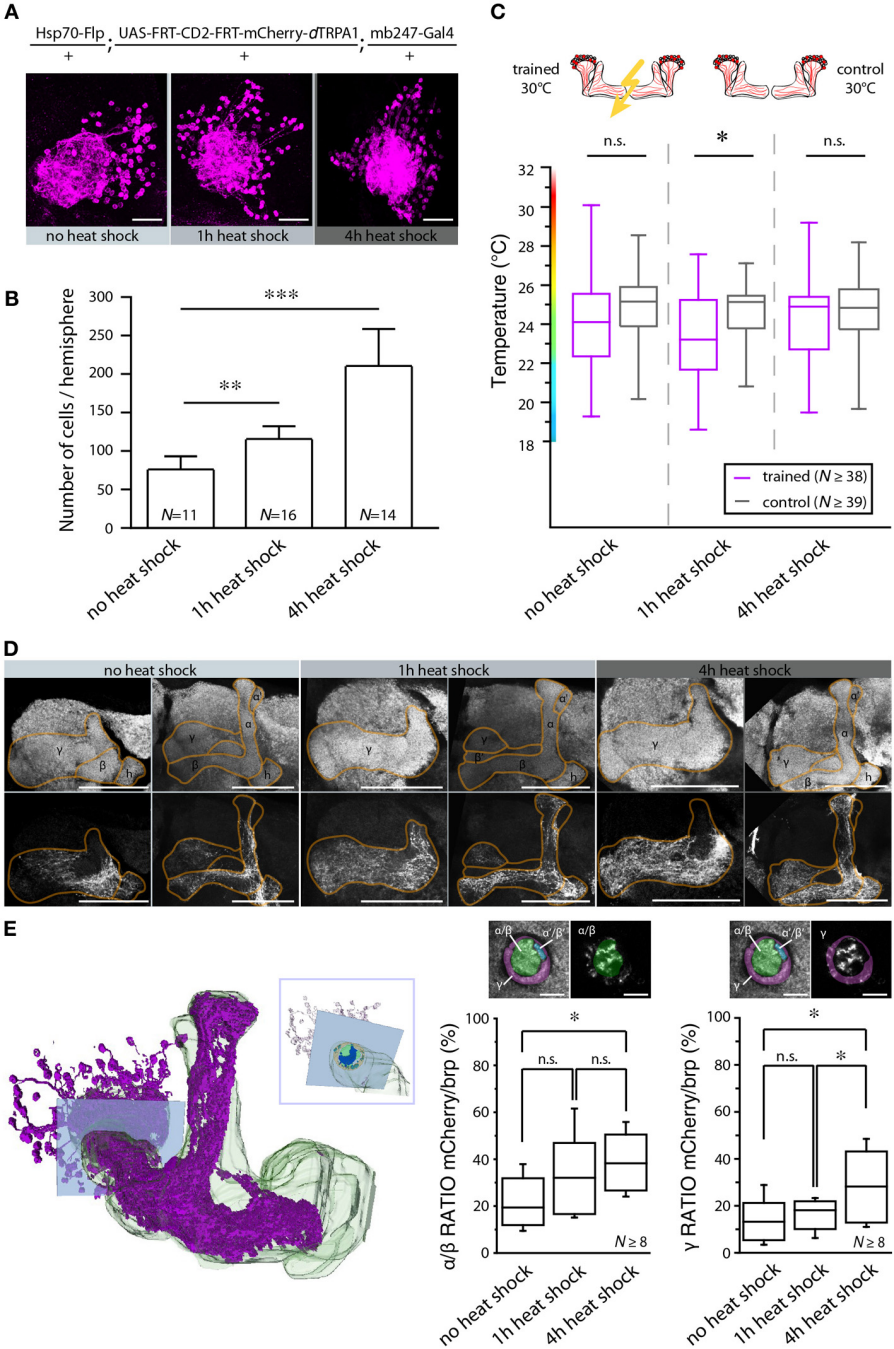
**The number of activated Kenyon cells affects the learning ability. (A)** Representative images of mushroom body calyces from flies receiving no heat shock, 1 or 4 h of heat shock during development to induce the expression of mCherry-*d*TRPA1. Scale bars = 30 μm. **(B)** Prolonging the heat shock during development causes an increase in the number of Kenyon cells expressing mCherry-*d*TRPA1 (^**^p < 0.01; ^***^p < 0.001. One-Way ANOVA). Bars indicate means ± SD. **(C)** Temperature preference of flies that express mCherry-*d*TRPA1 in ensembles of Kenyon cells and that have been treated at 30°C with electric shocks (“trained”) in comparison to flies that have not received electric shocks (“control”). The three groups differ in the duration of the gene expression induction and the number of Kenyon cells expressing mCherry-*d*TRPA1, as indicated in **(B)**. The graphs indicate medians, interquartile ranges, minimum and maximum values. A significant shift in temperature preference during the test situation is detected only in animals that have received a 1 h induction of gene expression during development when compared to control flies (*p* < 0.05). There is no significant difference in temperature preference in flies that have not received any heat shock (*p* > 0.1) or that have received a 4 h heat shock during development (*p* > 0.9) compared to the respective controls. Statistical test: bonferroni-corrected Mann-Whitney-U-test. (^*^p < 0.05). **(D)** Representative images of α/β- and γ-lobe mushroom body lobes of flies that have received no heat shock, 1 or 4 h of heat shock during development to induce the expression of mCherry-dTRPA1. Scale bars = 50 μm. **(E)** Left: 3D reconstruction of the mushroom bodies and Kenyon cells expressing mCherry-*d*TRPA1 (4 h heat-shock during development). The inlet indicates a focal plane (blue rectangle) at a region of the peduncle in which α/β- and γ –lobes can be anatomically differentiated. Right: quantification of area within the subregions of the peduncle that shows mCherry-*d*TRPA1 expression normalized to the total area determined by anti-bruchpilot (brp) staining. Prolonging the heat shock during development causes an increase in the relative area occupied by Kenyon cells expressing mCherry-*d*TRPA1 within the focal plane (^*^p < 0.05; One-Way ANOVA, *N* ≥ 8 mushroom bodies from 5 to 7 animals). Scale bars = 10 μm.

**Figure 10:**
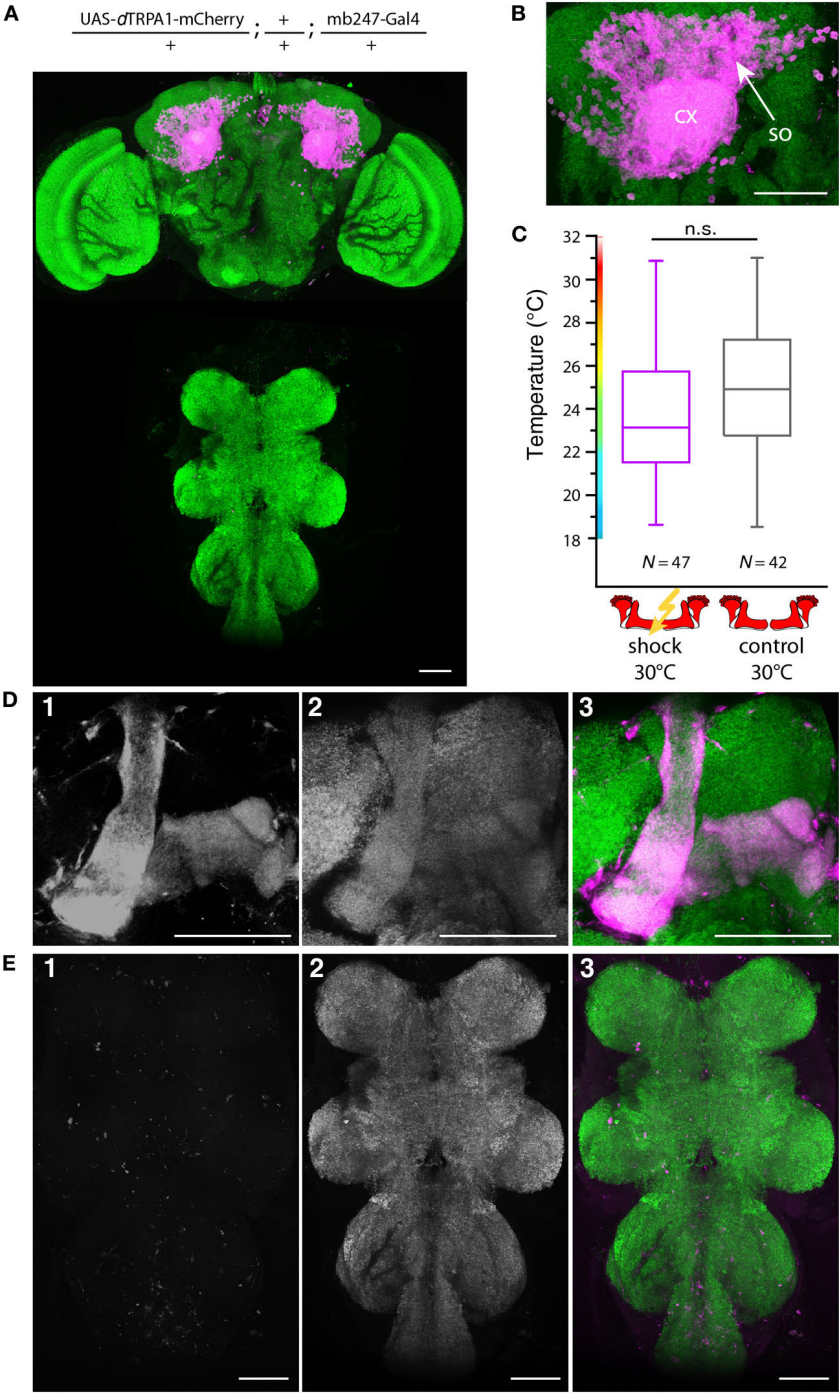
**Artificial activation of the majority of α/β- and γ-lobe Kenyon cells simultaneously with electric shocks does not induce learning. (A)** *d*TRPA1-mCherry expression in the brain and thoracic ganglion using the driver mb247-Gal4 **(B)** Magnification of the Kenyon cells somata (so) and the mushroom body calyx (cx). Scale bar = 30 μm. **(C)** Temperature preferences of trained and control flies that express *d*TRPA1-mCherry in Kenyon cells under control of mb247-Gal4. Box plots indicate medians, interquartile ranges, minimum and maximum values. Flies trained at 30°C with electric shocks showed a temperature preference that was not significantly different (*p* > 0.08; Mann-Whitney-U-test) to control animals that did not receive electric shocks. **(D)** Mb247-Gal4-induced expression of *d*TRPA1-mCherry **(1)** is restricted to α/β- and γ-lobes. Panel **(2)** shows anti-bruchpilot immunestaining as a neurpil marker. Panel **(3)** shows the overlay (magenta: mCherry, green: bruchpilot). **(E)** No expression of *d*TRPA1-mCherry in the thoracic ganglion is detectable **(1)**. Neuropils are stained with anti-bruchpilot antibody **(2)**. Panel **(3)** shows the overlay (magenta: mCherry, green: bruchpilot). Scale bars = 50 μm.

In *Drosophila* and other insects, odor stimuli induce neuronal activity in a relatively small number of Kenyon cells out of large neuronal populations, a principle of information encoding commonly referred to as “sparse coding” (Perez-Orive et al., 2002; Szyszka et al., 2005; Jortner et al., 2007; Ito et al., 2008; Murthy et al., 2008; Turner et al., 2008; Luo et al., 2010; Honegger et al., 2011). We tested whether the actual number of Kenyon cells that are activated affects the efficiency of associative learning and memory retrieval. We made use of the heat shock promoter to control flippase expression by means of various heat shock durations. The number of Kenyon cells expressing mCherry-*d*TRPA1 increased with increasing heat shock duration (Figures 9A,B,E). The Kenyon cells expressing mCherry-*d*TRPA1 project to all lobes included in the mb247-Gal4 line, i.e., α/β- and γ-lobes (Figures 9D,E). Flies that expressed mCherry-*d*TRPA1 in 76 ± 17 (mean ± SD) Kenyon cells did not show any associative learning (Figure 9C). However, flies that expressed mCherry-*d*TRPA1 in 116 ± 17 (mean ± SD) showed a significant temperature preference shift in trained flies (Figure 9C). These results demonstrate that learning induced through artificial activation of Kenyon cells, coincident with an electric shock, requires a minimum number of Kenyon cells. However, a further increase in the number of Kenyon cells that activated by mCherry-*d*TRPA1 to 211 ± 48 (mean ± SD) (Figure 9C), or by expressing *d*TRPA1-mCherry in large proportions of the α/β and γ lobes using mb247-Gal4 (Figures 10A,B,D,E), did not result in any significant learning (Figure 10C). Associative learning by ensembles of Kenyon cells depends, therefore, both on a minimum and a maximum number of Kenyon cells.

## Discussion

Decades of research have led to the current concept that the mushroom body of insects plays a critical role in associative olfactory learning (Heisenberg, 2003; Davis, 2005; Fiala, 2007). Our work contributes to this concept in two aspects. First, we show for the first time that the excitation of Kenyon cell ensembles in coincidence with an electric shock is sufficient and causative to induce an associative memory. Second, our data provide a concept how a memory stored in Kenyon cell ensembles is retrieved. The avoidance response the animals perform after training relies on a closed feedback-loop between the animals' behavioral action and the resulting reactivation (or its avoidance) of Kenyon cells. The subsets of Kenyon cells that are artificially activated in our experiments were determined by the mb247-Gal4 line (Zars et al., 2000), which drives gene expression in Kenyon cells of the α/β- and γ-lobes, but not of the α'/β '-lobes. For olfactory learning *rutabaga* expression and D1-like dopamine receptor DopR expression is required in the γ-lobes only (Zars et al., 2000; Qin et al., 2012), which points toward a predominant role of this Kenyon cell subpopulation for olfactory associative learning and short-term memory formation. The α/β-lobes have also been implicated in the retrieval of olfactory short-term memory (McGuire et al., 2001; Krashes et al., 2007). The role of α'/β '-lobes in associative odor learning remains unclear since synaptic transmission from neurons of the α'/β '-lobes have been reported to be required for the acquisition and the consolidation of an olfactory short-term memory (Krashes et al., 2007), but genetic rescue of the *rutabaga* mutation in α'/β '-lobes does not restore short-term memory (Blum et al., 2009). Our data suggest that learning can take place in the absence of α'/β '-lobe neuron activity, suggesting that these neurons are not required for the acquisition and retrieval of an associative memory in our paradigm. This does, however, not exclude the possibility that Kenyon cells of α'/β '-lobes can exhibit an equivalent function. We find, however, that learning induced through artificial activation of Kenyon cells, coincident with an electric shock, requires a minimum and a maximum number of Kenyon cells. This is in accordance with the anatomy and physiology of mushroom body output neurons, which suggests an integration of the synaptically-weighted and summed output across large populations of Kenyon cells (Cassenaer and Laurent, 2007, 2012; Séjourné et al., 2011). Interestingly, the number of Kenyon cells that are effective in inducing an associative memory (~5%) matches the reported number of Kenyon cells that are activated by odors (Turner et al., 2008; Honegger et al., 2011) and apparently reflects a range of sparseness that is optimized not only for information storage (coding), but also for memory readout (decoding). To which extend our experimental approach using an artificial activation of neurons indeed recapitulates the processes of associative learning of a natural odor stimulus remains to be investigated. However, our finding that a memory engram can be induced in sparsely activated ensembles of Kenyon cells shows striking parallels to reports involving mice, in which random ensembles of cortical neurons of different brain areas were optogenetically activated, together with a reward or a punishment (Huber et al., 2008; Choi et al., 2011; Liu et al., 2012). Here, the animals also learned to approach or avoid actively a reactivation of those cells that were optogenetically stimulated in coincidence with the punishment or reward, respectively (Choi et al., 2011). This situation is similar to our finding in which the retrieval of the aversive memory is characterized by a closed feedback loop between the behavioral action and, as a consequence, the activity of the trained Kenyon cells. Hence, despite the evolutionary distance between mammals and insects their neuronal networks share common principles in information processing and memory formation.

### Conflict of interest statement

The authors declare that the research was conducted in the absence of any commercial or financial relationships that could be construed as a potential conflict of interest.
